# Modulating Nanozyme‐Based Nanomachines via Microenvironmental Feedback for Differential Photothermal Therapy of Orthotopic Gliomas

**DOI:** 10.1002/advs.202204937

**Published:** 2022-11-27

**Authors:** Na Yin, Yinghui Wang, Ying Huang, Yue Cao, Longhai Jin, Jianhua Liu, Tianqi Zhang, Shuyan Song, Xiaogang Liu, Hongjie Zhang

**Affiliations:** ^1^ State Key Laboratory of Rare Earth Resource Utilization Changchun Institute of Applied Chemistry Chinese Academy of Sciences Changchun Jilin 130022 China; ^2^ School of Applied Chemistry and Engineering University of Science and Technology of China Hefei Anhui 230026 China; ^3^ Department of Neurosurgery The First Hospital of Jilin University Changchun Jilin 130061 China; ^4^ Department of Radiology The Second Hospital of Jilin University Changchun Jilin 130041 China; ^5^ Department of Chemistry National University of Singapore Singapore 117543 Singapore; ^6^ Department of Chemistry Tsinghua University 100084 Beijing China

**Keywords:** inflammation, nanomachines, nanozyme, orthotopic glioma, photothermal therapy

## Abstract

Gliomas are common and refractory primary tumors closely associated with the fine structures of the brain. Photothermal therapy (PTT) has recently shown promise as an effective treatment for gliomas. However, nonspecific accumulation of photothermal agents may affect adjacent normal brain structures, and the inflammatory response induced during PTT may result in an increased risk of brain tumor recurrence or metastasis. Here, the design and fabrication of an intelligent nanomachine is reported based on Gd_2_O_3_@Ir/TMB‐RVG29 (G@IT‐R) hybrid nanomaterials. These nanomaterials enable tumor‐specific PTT and eliminate inflammation to protect normal brain tissue. The mechanism involves the rabies virus glycopeptide‐29 peptide (RVG29) passing through the blood–brain barrier (BBB) and targeting gliomas. In the tumor microenvironment, Ir nanozymes can act as logic control systems to trigger chromogenic reaction amplification of 3,3′,5,5′‐tetramethylbenzidine (TMB) for tumor‐specific PTT, whereas in normal brain tissues, they scavenge reactive oxygen species (ROS) generated by poor therapy and function as protective agents. Autophagy inhibition of Gd_2_O_3_ enables excellent photothermal therapeutic effects on orthotopic gliomas and protection against inflammation in normal cells. The results of this study may prove useful in developing highly efficient nanomedicines for glioma treatment.

## Graphical abstract

The Ir nanozyme‐based nanomachines can feedback‐regulate the functional states according to microenvironments to realize glioma‐specific photothermal therapy (PTT) and inflammation clearance to normal cells. Tail‐vein‐injected nanomachines can effectively cross the blood–brain barrier and target gliomas with excellent inhibition of orthotopic glioma with the assistance of autophagy inhibition.

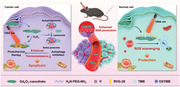

## 1 Introduction

Photothermal therapy (PTT) has emerged as a safe therapeutic modality that can generate localized thermal damage in the target area by controlling the photoirradiation parameters, thereby reducing side effects.^[^ ^1^ ^]^ PTT has achieved great success in subcutaneous tumor models due to its inherent advantages.^[^ ^2^ ^]^ Unlike subcutaneous tumor models, the primary tumor has a more diverse site and depth, as well as a more complicated microenvironment. Primary glioma is one of the lethal forms of brain cancers because it is aggressive and highly invasive.^[^ ^3^ ^]^ It is densely surrounded by adjacent healthy cells such as neurons, microglia, stromal cells, and astrocytes that support unparalleled levels of cognition and behaviors.^[^ ^4^ ^]^ Thus, for primary brain glioma, the photothermal effect inevitably damages these normal cells because of the macroscopic range of usual light sources.^[^ ^5^ ^]^ To alleviate the side effects of PTT on normal tissues, many strategies have been proposed.^[^ ^6^ ^]^ Shi and co‐workers constructed acidity/reducibility‐induced photothermal conversion materials for tumor‐specific photo‐hyperthermia.^[^ ^7^ ^]^ Tan and co‐workers developed a “nitric oxide (NO)/acidity” dual‐stimuli‐responsive PTT and achieved cancer therapy specificity with reduced toxic side effects in the 4T1 xenograft tumor model.^[^ ^8^ ^]^ Therefore, it is a promising strategy to develop tumor microenvironment‐responsive photothermal agents that can specifically perform PTT on brain tumors in situ and have low toxicity to normal cells.^[^ ^9^ ^]^

Apart from the above issues, inflammatory processes and upregulation of inflammatory factors are often seen in photothermal tumor ablation during PTT,^[^ ^10^ ^]^ which play a central role in various neurological diseases, such as Alzheimer's and Parkinson's diseases.^[^ ^11^ ^]^ In addition, the excessive inflammatory response may incur permanent injury to normal brain tissue around brain tumor lesions and increase the risk of recurrence or metastasis of brain tumors.^[^ ^12^ ^]^ To circumvent the above problems, photothermal agents with anti‐inflammatory effects may be of great significance for the treatment of aggressive gliomas.^[^ ^13^ ^]^ Therefore, it is imperative to develop intelligent nanosystems for differential PTT that can perform different functions, such as in situ destruction of tumor cells by the photothermal effect and protection of normal cells.

Nanoscale “man‐made” machines (nanomachines) may be programmed with specific tasks to automatically respond to the changing environment and switch their programs to execute corresponding functions.^[^ ^14^ ^]^ Nanozymes, nanomaterials with enzyme‐like activities, are promising candidates for fabricating nanomachines because of their excellent performances, including flexible designs, controllable enzyme‐like activities, and environmental resistance.^[^ ^15^ ^]^ Nanozymes with multienzyme‐mimicking activities can sensitively capture the signal changes in the physiological environment and change the enzyme activities with the environment.^[^ ^16^ ^]^ Logic control nanosystems can modulate the “ON” and “OFF” states based on the microenvironment, making them ideal for differential PTT.^[^ ^17^ ^]^

**Scheme 1:**
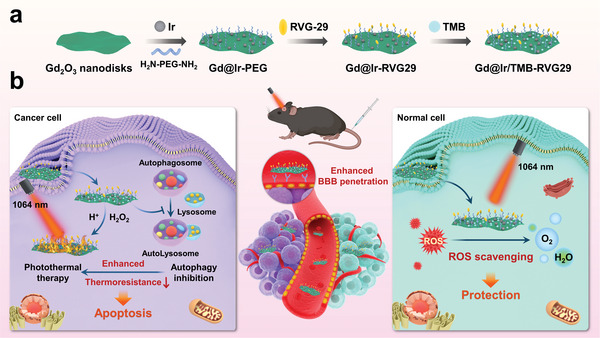
Schematic formation of a) the Gd_2_O_3_@Ir/TMB‐RVG29 (G@IT‐R) nanomachines. b) G@IT‐R nanomachines interacting with cancer cells and normal cells to enable a differential PTT strategy.

Building on these ideas, we designed an intelligent nanomachine (Gd_2_O_3_@Ir/TMB‐RVG29, abbreviated as G@IT‐R) by in situ growing Ir nanozymes and loading a pro‐photothermal agent, 3,3′,5,5′‐tetramethylbenzidine (TMB), on Gd‐based nanodisks modified with a brain‐targeting rabies virus glycoprotein‐29 peptide (RVG29), as shown in **Scheme** **1**. First, modification of RVG29 endows the nanomachine with good blood–brain barrier (BBB) permeability and further target gliomas.^[^ ^18^ ^]^ Ir nanozyme serves as a logic control system that modulates the “ON” state by the activators of the acid and high concentration of H_2_O_2_ in TME. Specifically, under the stimulation of TME, Ir nanozyme in the nanomachine mainly presents peroxidase (POD)‐like activity and further leads to a significant amplification of the chromogenic response of the pro‐photothermal agent TMB. In contrast, in normal tissues, the pro‐photothermal agent remains silent, and the nanomachine scavenges the ROS generated by the poor therapy and plays a protective role. Additionally, due to the paramagnetic properties of Gd_2_O_3_, the nanomachine could act as a specific contrast agent for magnetic resonance imaging (MRI), enabling the nanomachine to control the exogenous laser and monitor the treatment effect. More importantly, Gd_2_O_3_ nanodisks have been shown to inhibit autophagy and promote PTT in nanomachines.^[^ ^19^ ^]^ With these merits, the intelligent nanomachines achieve glioma‐specific therapy while alleviating adjacent the damage to normal brain tissues via a differential PTT strategy, which may be beneficial for future nanomedicine design.

## 2 Result and Discussion

### 2.1 Design and Synthesis of the G@IT‐R Nanomachines

**Figure 1:**
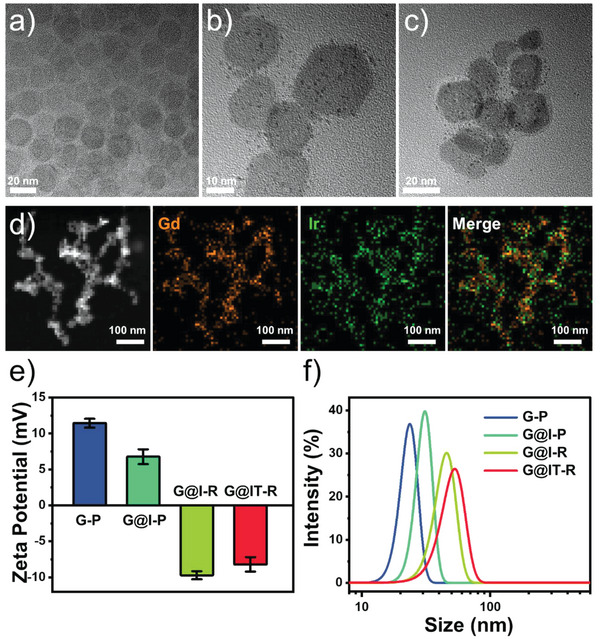
Characterizations of the nanomachines. a) TEM image of Gd_2_O_3_ nanodisks. b) TEM image of Gd_2_O_3_@Ir‐PEG NPs. c) TEM image of Gd_2_O_3_@Ir/TMB‐RVG29 (G@IT‐R). d) Scanning transmission electron microscopy (STEM) image and corresponding element mapping of G@IT‐R. e) Zeta potentials and f) hydrodynamic diameters of G–P, G@I‐P, G@I‐R, and G@IT‐R.

First, ultrathin Gd_2_O_3_ nanodisks were fabricated by thermal decomposition of gadolinium‐acetate precursors. Transmission electron microscopy (TEM) images of the as‐synthesized Gd_2_O_3_ exhibited a disk‐like morphology with a relatively uniform lateral dimension of ≈20 nm and excellent transparency (**Figure** **1**a).^[^ ^20^ ^]^ Interestingly, some Gd_2_O_3_ nanodisks assembled into a “face‐to‐face stack” on the TEM grids (Figure S1, Supporting Information). The thicknesses of the nanodisks are identical, ≈1.1 nm.^[^ ^21^ ^]^ The X‐ray diffraction (XRD) patterns showed the highly anisotropic nature of as‐synthesized Gd_2_O_3_ nanodisks, with broad (222) and (622) peaks (Figure S2, Supporting Information). The as‐prepared Gd_2_O_3_ nanodisks have {110} planes stacked along the diameter and {111} planes forming top and bottom surfaces.^[^ ^22^ ^]^ Polyvinyl pyrrolidone (PVP) and amine (polyethylene glycol) amine (H_2_N—PEG—NH_2_) were modified on the surface of ultrathin Gd_2_O_3_ nanodisks (Gd_2_O_3_–PEG, G–P) to improve their stability in physiological solutions and biocompatibility. Then, Ir nanodots were grown in situ on the surface of G–P. The TEM image of the obtained Gd_2_O_3_@Ir‐PEG (G@I‐P) clearly showed that Ir nanodots grew uniformly on the surface of the G–P nanodisk (Figure 1b). Subsequently, G@I‐P were further modified with RVG29 peptide (Gd_2_O_3_@Ir‐RVG29, G@I‐R) by the reaction with the hetero‐bifunctional crosslinkers so that they could simultaneously penetrate the BBB and target gliomas. Subsequently, pro‐photothermal agent TMB molecules were efficiently loaded on the surface of G@I‐R with an optimal loading amount of 17.9%, which was due to the high affinity to their hydrophobic domain of PVP.

The nanomachines G@IT‐R had a relatively small diameter of 20–30 nm (Figure 1c). The XRD pattern of G@IT‐R mainly showed the crystal structure of Gd_2_O_3_ nanodisks, as the ultrasmall Ir nanodots had no obvious XRD peak (Figures S2 and S3, Supporting Information). From the results of the elemental mapping images, Ir element was homogeneously distributed on the surfaces of Gd_2_O_3_ nanodisks, confirming that Ir nanodots grew uniformly on Gd_2_O_3_ nanodisks (Figure 1d). We recorded the corresponding zeta potential and hydrodynamic sizes of these nanoparticles (NPs) by dynamic light scattering (DLS) analysis. The zeta potential and hydrodynamic size of G@I‐P are ≈+6.7 ± 1.0 mV and 31 ± 3.5 nm, respectively (Figure 1e,f). After RVG29 modification, the zeta potential and hydrodynamic size changed greatly to −9.7 ± 0.6 mV and 45 ± 5.7 nm, indicating the successful modification of RVG29. After loading TMB in G@I‐R, the hydrodynamic size changed slightly, which was also confirmed by the variation in zeta potential from −9.7 ± 0.6 to −8.2 ± 1.0 mV. Moreover, G@IT‐R nanomachines were dispersed in phosphate‐buffered saline (PBS) solution and 10% fetal bovine serum (FBS) for 72 h, and the hydrodynamic size did not change significantly, indicating their outstanding colloidal stability (Figure S4, Supporting Information).

### 2.2 In Vitro Logic Control System of the G@IT‐R Nanomachines

**Figure 2:**
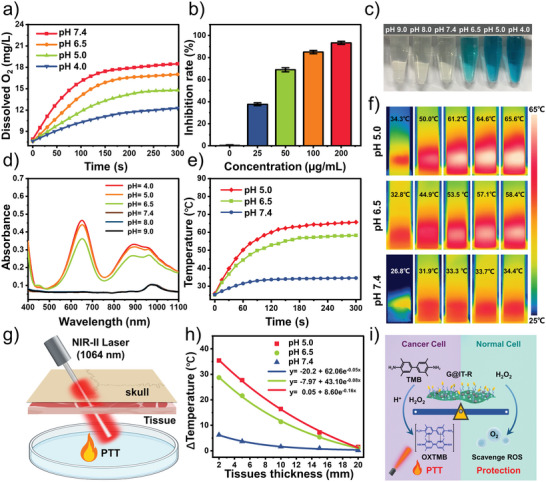
In vitro properties of nanomachines. a) The O_2_ concentration variations treated with G@IT‐R at pH 7.4, 6.5, 5.0, and 4.0 with H_2_O_2_ solutions (100 µm). b) ABTS• scavenging capacity of G@IT‐R at indicated concentration. c) The color evolution and d) UV–vis spectra of G@IT‐R in different pH buffer solutions with H_2_O_2_ solutions (100 µm). e) Real‐time temperature and f) infrared (IR) thermal images of G@IT‐R in pH 5.0, 6.5, and 7.4 PBS buffers with H_2_O_2_ solutions (100 µm), 1064 nm laser irradiation. g) Schematic illustration of intracranial deep‐tissue NIR‐II PT of G@IT‐R. h) Temperature decay of G@IT‐R with tissue depth at different pH conditions under 1064 nm laser irradiation (1.0 W cm^−2^). i) Illustration of the principle operation of the logic control systems in G@IT‐R nanomachines.

Nanozymes with a catalase (CAT)‐like capacity are widely used to reduce H_2_O_2_‐induced cellular oxidative damage. We first explored the CAT‐like activity of G@IT‐R using a dissolved oxygen electrode.^[^ ^23^ ^]^ After adding H_2_O_2_ to the solution of G@IT‐R NPs, the produced gas was detected as O_2._ The oxygen produced gradually decreases as pH in the solution decreases, implying a concomitant decrease in the CAT‐like enzyme activity of G@IT‐R nanomachines (**Figure** **2**a). The antioxidative activity of G@IT‐R was verified by the 2,2′‐azino‐bis(3‐ethylbenzothiazoline‐6‐sulfonate) (ABTS) radical assay. As shown in Figure 2b, more than 80% of ABTS radicals were eliminated with 100 µg mL^−1^ of G@IT‐R. Then, the POD‐like activity of G@IT‐R was investigated in different pH environments through discoloration.^[^ ^24^ ^]^ As shown in Figure 2c, the discoloration of G@IT‐R incubated with H_2_O_2_ presented a high sensitivity toward pH (ranging from 4.0 to 9.0). The strong absorption peak at 650 nm was assigned to oxidized TMB, indicating that G@IT‐R mimicked the activity of POD (Figure 2d).^[^ ^25^ ^]^ In the acidic condition, the H_2_O_2_/G@IT‐R solution presented a strong absorption, but the absorption in the neutral or slightly basic condition (pH ≥ 7) almost disappeared. These results confirmed that G@IT‐R exhibited only CAT‐mimetic activity at pH 7.4, whereas with an increase in acidity, the CAT‐like activity reduced and the POD‐like activity was dramatically stimulated at the same time.

The broadband absorptivity feature of G@IT‐R from the visible to the second near‐infrared (NIR‐II) region allowed it to be used as a photothermal agent after reaction with H_2_O_2_ (Figure 2d). As shown in Figure 2e, the temperature easily increased to above 55 °C with H_2_O_2_‐incubated G@IT‐R solutions at pH 5.0 and 6.5 after irradiation with a 1064 nm laser (1.0 W cm^−2^, 5 min).^[^ ^26^ ^]^ However, at pH 7.4, no significant changes in temperature were observed. IR thermal camera images further verified these results (Figure 2f). Notably, the skull and brain parenchyma could affect the NIR‐II laser penetration. The penetrating ability was tested using a brain‐mimicking phantom covered with a layer of mouse skull and chicken breast muscles of different thicknesses (Figure 2g). As shown in Figure 2h, the temperature of H_2_O_2_‐activated G@IT‐R solutions decreased with increasing tissue depth under 1064 nm irradiation (1.0 W cm^−2^, 10 min). The temperature increased by about 20 °C with a covered tissue depth of 5 mm at pH 6.5, which was sufficient for PPT according to the 10–13 °C threshold of temperature increases for in vivo PTT.^[^ ^27^ ^]^ The working principle of a logic control system in G@IT‐R nanomachines is summarized in Figure 2i. G@IT‐R exhibited different enzyme activities in different microenvironments, which allowed them to simultaneously achieve specific PTT for tumor and protective effects on normal cells.

### 2.3 In Vitro BBB‐Penetrating and Glioma‐Cell Uptake of the G@IT‐R Nanomachines

**Figure 3:**
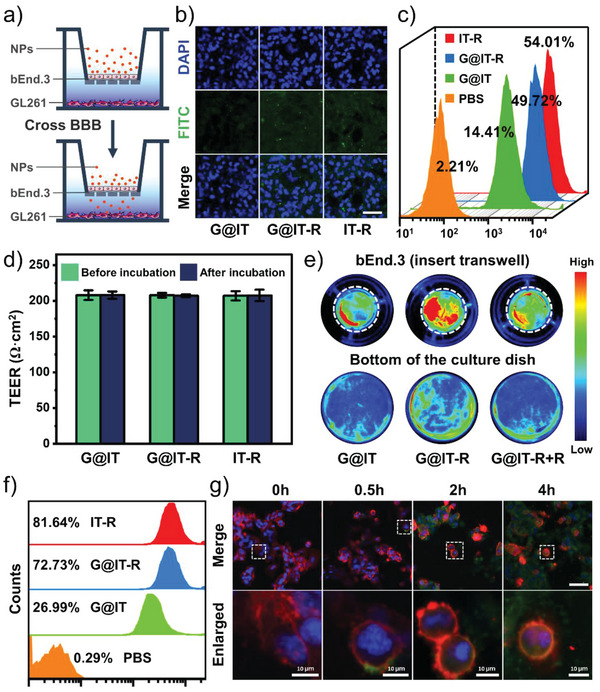
a) The schematic illustration of in vitro BBB model. b) CLSM and c) quantitative flow cytometric analysis showing the uptake of nanoparticles by GL261 cells in the bottom well after incubation with FITC‐labeled IT‐R, G@IT‐R, and G@IT. Scale bar: 50 µm. d) The TEER of the bEnd.3 monolayer before and after incubation with different treatments. e) Fluorescence images of the insert transwell and the bottom chamber after 4 h of incubation with different nanodrugs. White circles represent inset transwell. f) Quantitative flow cytometric analysis of cellular uptake of GL261 cells after co‐incubation with different nanodrugs for 4 h. g) CLSM showed cellular uptake of FITC‐labeled G@IT‐R by GL261 cells at indicated time points. For each panel in panel (g), cell nuclei are stained using 4′,6‐Diamidine‐2′‐phenylindole dihydrochloride (DAPI) (blue), nanoparticles are labeled with FITC, and cell membranes are stained with red dye. Scale bar: 50 µm.

The BBB blocks the entry of most drugs into the central nervous system (CNS), which limits the entry of these drugs through the circulatory system to the brain and limits the prevention, diagnosis, and treatment of brain disorders.^[^ ^28^ ^]^ RVG 29 peptides can specifically bind the nicotinic acetylcholine receptor (nAchR) that is extensively located on the extracellular surface of glioma cells and brain microvascular endothelial cells, giving G@IT‐R nanomachines the potential to overcome the BBB and subsequently internalize into glioma cells.^[^ ^18^, ^29^ ^]^ First, the cytotoxicity of G@IT‐R against bEnd.3 cells (mouse brain microvascular endothelial cells, the main component of BBB) and GL261 cells was evaluated by CCK8 assay (Figure S5, Supporting Information). When incubated with G@IT‐R nanomachines at a concentration of 200 µg mL^−1^, the cells maintained over 80% viability, indicating that G@IT‐R had good biosafety and can be used for evaluating the ability of RVG29‐mediated BBB permeability. The in vitro BBB model was constructed with a transwell system comprising an insertion chamber containing brain microvascular endothelial (bEnd.3) cells and a bottom chamber containing glioma tumor (GL261) cells (**Figure** **3**a). Transendothelial electrical resistance (TEER) is widely used to assess cellular barrier tightness. When the TEER value was ≥200 Ω cm^2^, the in vitro BBB model can be considered as a tight junction as the in vivo BBB and could be used for subsequent experiments. The fluorescence marker fluorescein 5‐isothiocyanate (FITC) was used to label G@IT‐, G@IT‐R‐, and RVG29‐modified Ir nanodots with TMB (IT‐R), and separately added to the apical chamber. After 4 h of incubation, we investigated the fluorescence signals of GL261 cells in the bottom well by confocal laser scanning microscopy (CLSM). Compared with G@IT, G@IT‐R exhibited a much stronger fluorescence signal, indicating the importance of RVG29 surface modification (Figure 3b). IT‐R exhibited a fluorescence signal almost as strong as G@IT‐R. These results were further confirmed by flow cytometry, which demonstrated that RVG29 could efficiently promote the penetration of G@IT‐R and IT‐R through the BBB (Figure 3c). No obvious changes of TEER were detected after incubation with these nanoparticles, indicating that these nanoparticles did not disrupt the integrity of the bEnd.3 cell monolayer (Figure 3d).

We further performed fluorescence imaging of the whole insert chamber (after wash) and the bottom chamber after different treatments. As shown in Figure 3e, after incubating with FITC‐labeled G@IT‐R for 4 h, the insert and bottom chambers exhibited much higher fluorescence signals than those after incubation with FITC‐labeled G@IT. However, this difference disappeared after pretreating the bEnd.3 cell monolayer with free RVG29 peptide, which implied that the specific interaction between RVG29 and bEnd.3 cell could enhance the penetration of G@IT‐R across the BBB mimetic cell monolayer. RVG29 may not only facilitate BBB transport but also mediate the subsequent cellular uptake. Therefore, whether RVG29 can increase the uptake of nanodrugs in GL261 cells was verified by flow cytometry. The fluorescence signals of GL261 cells treated with G@IT‐R were more than 40% higher than those cells exposed to G@IT (Figure 3f). This confirmed that the modification of RVG29 peptide could efficiently promote the cellular uptake of GL261. The CLSM images in Figure 3g show cellular uptake of G@IT‐R after being co‐incubated with GL261 cells for different times (0, 0.5, 2, and 4 h). The entire process of G@IT‐R nanomachines’ entry into the GL261 cells can be clearly seen in the representative enlarged images of different incubation times; after 0.5 h of incubation, the G@IT‐R nanomachines were attached to the cell membrane surface, and after 2 h of incubation the nanomachines were uptaken by the cells and colocalized with the cytoplasm, then the uptake increased after 4 h.

### 2.4 Differential Photothermal Therapeutic Effect in Glioma Cells

**Figure 4:**
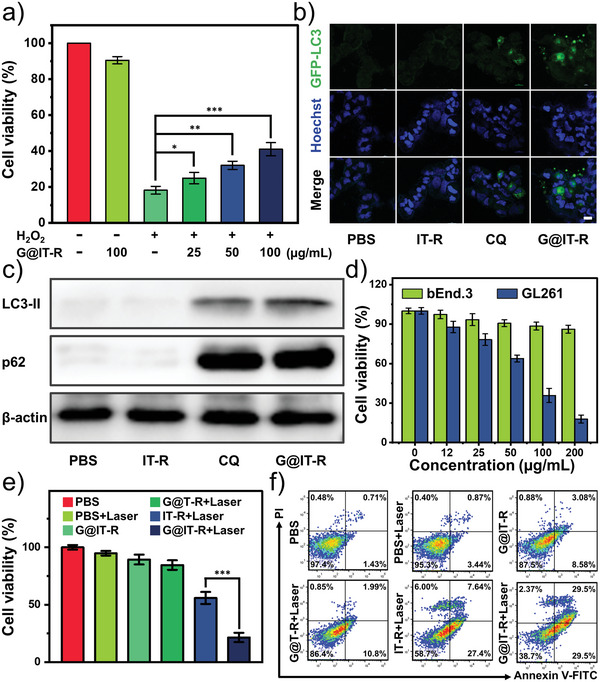
a) Viability of bEnd.3 cells incubated with different treatments. H_2_O_2_ (300 µm). b) Accumulation of GFP‐LC3 puncta in GL261 after treatment with IT‐R (10 µg Ir mL^−1^), G@IT‐R (10 µg Ir mL^−1^), and CQ (2 × 10^−5^ m) for 12 h. The distribution of GFP‐LC3 puncta was observed with a confocal laser scanning microscope. Scale bar: 20 µm. c) The protein expression of LC3‐II and p62 in GL261 cells of each group was determined by western blot analysis. d) Viability test of photothermal destruction of bEnd.3 cells (cultured in pH = 7.4 DMEM without H_2_O_2_) and GL261 cells (cultured in pH = 6.5 DMEM with 100 µm H_2_O_2_) after 5 min laser irradiation at 1064 nm (0.5 W cm^−2^). e) Viability of GL261 cells incubated with different treatments after 24 h. Data are expressed as mean ± standard deviation (SD; *n* = 6). f) Fluorescein‐annexin V and propidium iodide (PI) staining assays of GL261 cells after various treatments.

In nature, CAT has been used to protect cells from oxidative damage by H_2_O_2_. However, the cells in pathological situations may lose the ability to decompose H_2_O_2_, resulting in severe protein and DNA damage.^[^ ^30^ ^]^ Many studies have focused on the development of highly efficient ROS scavengers that have made tremendous progress in brain disorders such as stroke and cerebral ischemia‐reperfusion injury.^[^ ^23^, ^31^ ^]^ Therefore, the antioxidant and anti‐inflammatory effects of G@IT‐R nanomachines were evaluated on normal neuronal cells (bEnd.3). We used H_2_O_2_ (300 µm) to stimulate cells and induce oxidative stress. The cell viability was 90% after treatment with G@IT‐R alone, while it decreased to 18% when exposed to H_2_O_2_ alone. Moreover, it could be found that G@IT‐R protected the cells against H_2_O_2_‐induced death in a dose‐dependent manner (**Figure** **4**a).

Autophagy refers to a cellular process involving the degradation and recovery of damaged organelles and other components. Autophagy can protect tumor cells from heat‐induced damage by promoting heat‐induced damage repair.^[^ ^32^ ^]^ Currently, more research is focusing on the combination of autophagy inhibitors and PTT. However, clinically used autophagy inhibitors such as chloroquine (CQ) may cause systemic toxicity.^[^ ^33^ ^]^ Nanoparticles used as autophagy inhibitors hold great promise.^[^ ^34^ ^]^ Therefore, we first explored whether G@IT‐R could influence the autophagy of tumor cells. Microtubule‐associated protein 1 light chain 3 (LC3) is a crucial autophagy‐related protein. LC3‐II as a standard autophagosome marker is commonly used to monitor autophagic activity, and its number correlates positively with the number of autophagosomes. Adenovirus expressing green fluorescent protein‐tagged LC3B (GFP‐LC3B) was transfected into GL261 cells. CLSM images showed that many green punctate dots formed after incubation with autophagy inhibitor CQ and G@IT‐R demonstrating the recruitment of LC3 for autophagosome formation. The LC3 in the cells treated with PBS and IT‐R was diffusely distributed throughout the cytoplasm (Figure 4b). Autophagosome accumulation can be caused by autophagy activation in the upstream process or autophagic flux blockage in the later stage, which urgently prompts us to make a distinction between these two possibilities. We investigated the increased level of p62 to monitor the extent of blockade of autophagic flux using a western blot assay. As shown in Figure 4c and Figure S6 (Supporting Information), the expressions of both LC3‐II and p62 were significantly increased in GL261 cells treated with G@IT‐R and CQ in comparison with those treated with PBS and IT‐R. These results show that G@IT‐R can effectively inhibit the autophagy flux.

We subsequently investigated the feasibility of the nanomachine to achieve differential PTT through logical system recognition of the environment and subsequent control over the functional system. We assessed the cell viability of glioma cells and normal cells after undergoing nanomachine and PTT. GL261 cells cultured in simulated TME (pH = 6.5 dulbecco's modified eagle medium (DMEM) and 100 µm H_2_O_2_) were killed in a dose‐dependent manner after treatment with G@IT‐R and 1064 nm laser (0.5 W cm^−2^, for 5 min), demonstrating that G@IT‐R has efficient photothermal therapeutic ability for GL261 cells (Figure 4d). bEnd.3 cells cultured in a “healthy” intracellular environment (pH = 7.4 DMEM without H_2_O_2_) showed high cell viability when treated with 200 µg mL^−1^ of G@IT‐R and 1064 nm laser, indicating that G@IT‐R‐based PTT was highly specific to cells in simulated TME. We further evaluated the therapeutic effect of different groups on glioma GL261 cells, including PBS, PBS+Laser, G@IT‐R, IT‐R+Laser, G@T‐R+Laser, and G@IT‐R+Laser. The viabilities of the cells after treatment with PBS+Laser or G@IT‐R were 95% and 89%, respectively, illustrating that nanodrugs and 1064 nm laser with low power density alone were not cytotoxic. Compared with G@IT‐R+Laser, the cell viability of group G@T‐R+Laser was more than 80%, indicating that Ir nanodots with the effect of activating photothermal prodrugs are indispensable components in PTT. Compared with the IT‐R+Laser group, the GL261 cells treated with G@IT‐R+Laser had a low cell viability of 21.5%, implying that the photothermal therapeutic effect was enhanced by the nano‐autophagy inhibitor Gd_2_O_3_ (Figure 4e). The similar cytotoxicity results were observed in a live/dead cell staining analysis (Figure S7, Supporting Information). Moreover, apoptosis analysis of these cells with different treatments further proved the above results, indicating the great potential of our designed nanomachines in the treatment of gliomas (Figure 4f).

### 2.5 In Vivo Evaluation of MRI in Orthotopic Gliomas

**Figure 5:**
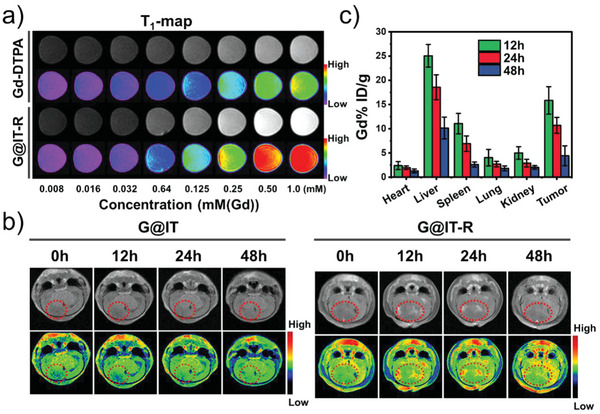
a) In vitro MRI *T*_1_‐map of Gd‐DTPA and G@IT‐R. b) Representative in vivo brains *T*_1_‐weighted MRI of intracranial GL261 glioma mice by tail vein injection with G@IT or G@IT‐R at different time points (the red circle indicates representative tumor area). c) The body distribution of Gd content after intravenous injection of G@IT‐R nanomachines at different time points.

As a noninvasive method, MRI can provide the anatomical structure and high spatial resolution of brain tumors, such as gliomas.^[^ ^35^ ^]^ The super‐paramagnetism of Gd makes G@IT‐R a promising *T*_1_‐weighted MRI contrast agent. As shown in **Figure** **5**a, G@IT‐R showed a concentration‐dependent enhancement of the *T*_1_‐weighted MRI signal. The *r*_1_ relaxivity of G@IT‐R was 8.63 mm ^−1^ s^−1^, which was fourfold higher than that of clinically used gadopentetic acid (Gd‐DTPA) contrast agents (1.8 mm ^−1^ s^−1^) (Figure S8, Supporting Information). We further established the orthotopic glioma models to evaluate the potential of G@IT‐R as an MRI contrast agent for orthotopic gliomas in vivo. After intravenous injection (i. v.) of G@IT and G@IT‐R (10 mg Gd kg^−1^), MRI data were acquired at 0, 12, 24, and 48 h time points. In the MR images of Figure 5b, the most noticeable difference was observed at 12 h between cancer and adjacent brain tissues after i.v. injection of G@IT‐R, and then the signal became attenuated. At postinjection 48 h, the difference disappeared completely. In contrast, no significant MRI signal enhancement was observed after i.v. injection of G@IT. Significant differences in MR signal intensity of tumor areas in different groups were measured by quantitative analysis (Figure S9, Supporting Information). These results confirmed that RVG29‐mediated G@IT‐R crossing the BBB and targeting gliomas plays an important role for MRI in orthotopic gliomas. We next assessed the biodistribution of G@IT‐R and G@IT on glioma‐bearing mice by measuring the concentrations of Gd in dissected organs, including heart, lung, spleen, kidney, liver, and tumor at different time points using inductively coupled plasma mass spectrometry (ICP‐MS) (Figure 5c). The biodistribution of Gd showed similar results to MRI, i.e., the highest enrichment of G@IT‐R at the 12 h time point in orthotopic gliomas, and G@IT was not significantly enriched in the glioma (Figure S10, Supporting Information).

### 2.6 In Vivo Differential Photothermal Therapeutic Effect

**Figure 6:**
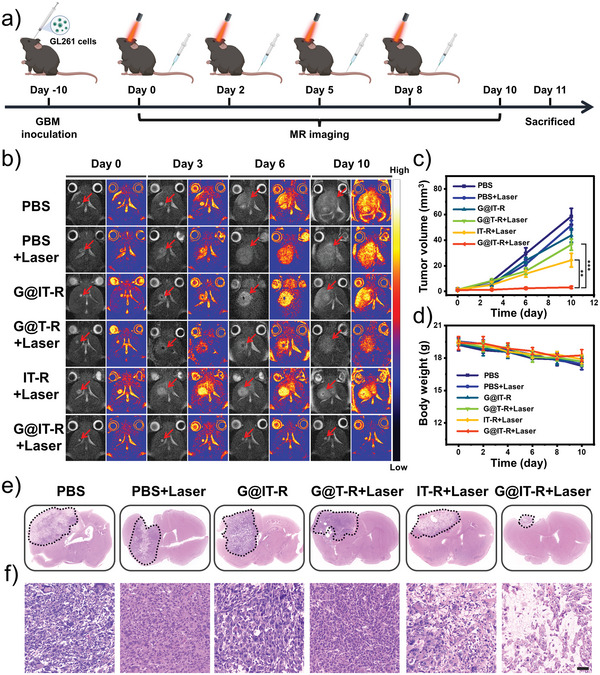
a) Schematic of the experimental design. b) *T*_2_‐weighted MRI images of the brains of mice with orthotopic gliomas in different groups reflecting tumor size (red arrow points to tumor area). c) Tumor volume of gliomas in each group. d) Average body weight of each group (*n* = 5). e) Histological analysis of mouse gliomas after different treatments. f) H&E‐stained gliomas slices in each group. Scale bar: 50 µm.

Encouraged by the desirable cellular anticancer efficacy of G@IT‐R, we evaluated the therapeutic efficacy in vivo in orthotopic glioblastoma models. About 10 days after cell inoculation, glioma‐bearing mice were randomly divided into six groups (*n* = 5) and treated with the following different treatments: 1) PBS, 2) PBS+Laser, 3) G@IT‐R, 4) G@T‐R+Laser, 5) IT‐R+Laser, and 6) G@IT‐R+Laser. Laser irradiation (1.0 W cm^−2^, 10 min) was carried out at 12 h postinjection. The tumor volume of orthotopic glioblastoma was dynamically monitored by MRI for 10 days (**Figure** **6**a). As shown in Figure 6b,c, the tumor volume in the PBS group increased significantly over time. Negligible inhibition of orthotopic gliomas was observed in the groups of “PBS+Laser” and “G@IT‐R,” indicating that alone laser or nanomachines failed to present a therapeutic effect. The G@T‐R+Laser treatment presented an insignificant antitumor effect because the absence of Ir nanozyme was unable to activate the photothermal prodrug. The tumor in the group “IT‐R+Laser” was slightly inhibited. In contrast, the growth of orthotopic glioma was nearly suppressed in the G@IT‐R+Laser groups, suggesting that autophagy inhibition greatly enhanced the PTT.^[^ ^36^ ^]^ Additionally, no obvious changes in the body weight of these mice were observed throughout the treatment period in all groups, indicating that the in vivo toxicity of these nanomaterials is negligible at the currently administered dose (Figure 6d). Histological hematoxylin and eosin (H&E) staining of representative brain sections of different groups is presented in Figure 6e, consistent with the above observations. The glioma in the “G@IT‐R+Laser” group was much smaller than those in the other five groups. Moreover, the glioma tumors of the PBS group had a much higher cell density, while the glioma tumor cells in the “G@IT‐R+Laser” group were the most damaged and displayed the lowest cell density.

**Figure 7:**
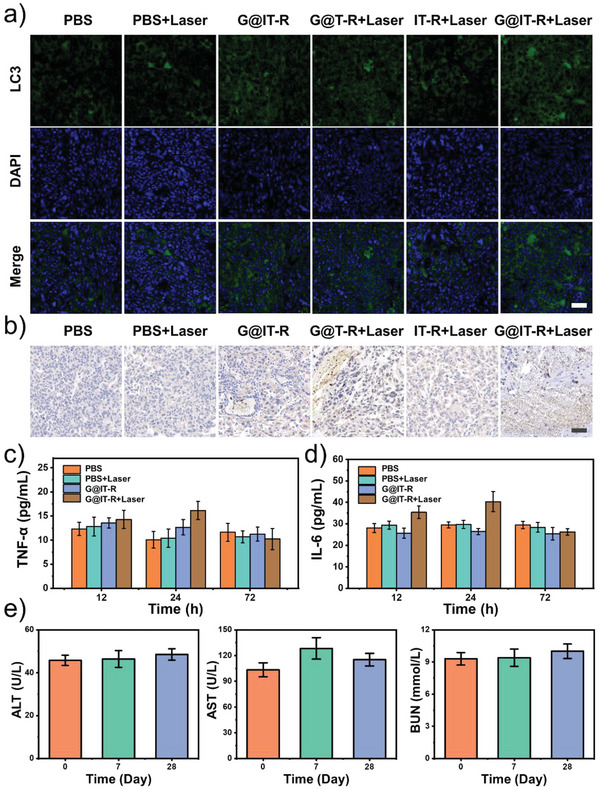
a) Representative immunofluorescence images of LC3 in tumor tissues of glioma‐bearing mice treated with different treatments. Scale bar: 50 µm. b) Immunohistochemistry analysis of p62 in tumor tissues from six groups of mice. Scale bar: 50 µm. c,d) Cytokine levels in sera of mice after various treatments. e) Blood biochemistry at different time points of healthy female C57BL/6J mice treated with G@IT‐R. Blood urea nitrogen (BUN), aspartate transaminase (AST), alanine transaminase (ALT). Data are represented as mean ± SD (*n* = 3).

To further confirm the changes in autophagy flux after different treatments, the expression of LC3 and p62 in orthotopic glioma sections was also assessed. The expression of LC3 and P62 was significantly upregulated in G@IT‐R+Laser groups compared with the PBS, PBS+Laser, and IT‐R+Laser groups (**Figure** **7**a,b). This proves that G@IT‐R, as an autophagic flux inhibitor, can effectively inhibit autophagy and assist PTT in gliomas.^[^ ^37^ ^]^

Cytokines including interleukin (IL)‐6 and tumor necrosis factor‐*α* (TNF‐*α*) were highly sensitive markers for acute inflammation.^[^ ^13a^ ^]^ To further validate the results of the nanomachines for clearing inflammation caused by thermal diffusion, we measured the IL‐6 and TNF‐*α* levels in sera of mouse at different times (Figure 7c,d). After 12 h of treatment, the concentration of TNF‐*α* and IL‐6 did not show a significant difference among these four groups. After 24 h of treatment, a slight increase in the level of TNF‐*α* and IL‐6 in the G@IT‐R+Laser group had been observed, while the cytokine levels in four groups were restored after 72 h. As reported, PTT without inflammatory scavenging will cause a several‐fold increase in cytokine levels.^[^ ^38^ ^]^ Only slightly increased cytokines during the treatment in G@IT‐R+Laser groups were mainly because of the obvious inflammation scavenging effect of Ir nanozymes. Therefore, these results strongly supported the anti‐inflammatory effect of G@IT‐R nanodrugs.

Subsequently, we collected major organs (heart, lung, spleen, kidney, and liver) of the mice at different time points after injection of G@IT‐R (dose: 10 mg  kg^−1^) for H&E staining to evaluate its biosafety (Figure S11, Supporting Information). No obvious tissue damage and adverse effects occurred after tail vein injection of G@IT‐R. Moreover, the blood parameters of mice treated with G@IT‐R for 7 and 28 days were not different from those of untreated animals (Figure 7e; Figure S12, Supporting Information).

## 3 Conclusion

We have elaborately developed a nanomachine based on nanozymes as a logic system to realize differential PTT. The modification of RVG29 endowed the G@IT‐R nanomachine with good BBB permeability and the ability to target gliomas, which is the basis of subsequent functions. By coactivating high concentrations of H_2_O_2_ and the acid in the TME, the nanomachine could activate the chromogenic reaction of the pro‐photothermal agent TMB to prevent nonspecific PTT. In normal tissues, the photothermal prodrug remained silent and the nanomachine functioned to scavenge excess ROS and further protect normal brain tissues. Moreover, the nanomachine was confirmed to act as an MRI contrast agent to monitor treatments and guide exogenous lasers. As a nanoautophagy inhibitor, it can enhance photothermal sensitization of cancer cells to improve efficacy. This differential PTT strategy provides a promising way to achieve satisfactory PTT for brain gliomas while alleviating toxic side effects on normal brain tissue. In addition, this strategy is likely to provide a new paradigm for the design of photothermal agents against brain cancers.

## Conflict of Interest

The authors declare no conflict of interest.

## Supporting information

Supporting Information

## Data Availability

The data that support the findings of this study are available from the corresponding author upon reasonable request.
