# Supplementary material for: Modulating Nanozyme‐Based Nanomachines via Microenvironmental Feedback for Differential Photothermal Therapy of Orthotopic Gliomas
Source: Adv Sci (Weinh). 2022 Nov 27;10(3):2204937. doi: 10.1002/advs.202204937 (PMC9875674; doi:10.1002/advs.202204937)
Supplement: Supplementary file 1 — Supporting Information [file ADVS-10-2204937-s001.pdf]

Supporting Information

Modulating Nanozyme-Based Nanomachines via Microenvironmental Feedback for Differential Photothermal Therapy of Orthotopic Gliomas

Na Yin, Yinghui Wang, * *Ying Huang, Yue Cao, Longhai Jin, Jianhua Liu, Tianqi Zhang, Shuyan Song, Xiaogang Liu and Hongjie Zhang* *

N. Yin, Y. Wang, Prof. S. Song, Prof. H. Zhang

State Key Laboratory of Rare Earth Resource Utilization, Changchun Institute of Applied Chemistry, Chinese Academy of Sciences, Changchun 130022, Jilin (China)

E-mail: yhwang@ciac.ac.cn; hongjie@ciac.ac.cn

N. Yin, Y. Wang, Y. Huang, Prof. S. Song, Prof. H. Zhang

University of Science and Technology of China, Hefei, Anhui, 230026 (China)

Prof. H. Zhang

Department of Chemistry, Tsinghua University, Beijing 100084 (China)

J. Liu, L. Jin, T. Zhang

Department of Radiology, The Second Hospital of Jilin University, Changchun 130041, Jilin (China)

Y. Cao

The First Hospital of Jilin University, Changchun, Jilin, 130061 (China)

Prof. X. Liu

Department of Chemistry, National University of Singapore, Singapore, 117543, Singapore.

METHOD

Materials. Oleic acid and Octadecene (>90%) were acquired from Aladdin Reagent, Ltd. (Shanghai, China). Gadolinium acetate hydrate (99.9%) was purchased from Thermo Fisher Scientific (Beijing, China). Iridium (Ⅲ) chloride trihydrate and PVP (Mw:24000-38000) were acquired from Macklin Biochemical Co., Ltd. (Shanghai, China). RVG29 peptides were purchased from China peptides Co., Ltd. (Suzhou, China). GL261 cells were purchased from Guangzhou Cellcook Biotech Co., Ltd. (Guangzhou, China). Calcein acetoxymethyl ester and propidium iodide were obtained from Sigma-Aldrich (America). Maleimide-[polyethylene glycol]-N-hydroxysuccinimide (MAL-PEG5000-NHS) was obtained from Xi'an ruixi Biological Technology Co., Ltd. (Xi'an, China). Transwell BD Matrigel was purchased from Corning (America).

Measurements. All powder X-ray diffraction patterns were obtained on a Powder X-ray Diffractometer (Bruker AXS, Germany). The average diameter of NPs fabricated was determined by Malvern Zetasizer Nano ZS90. The O_2 concentrations were measured by a portable dissolved oxygen meter (INESA Scientific Instrument Co., Ltd, Shanghai, China). The *in vitro* MR imaging ability were investigated by clinical MRI scanner (GE Discovery MR750 3.0 T). And *in vivo* MR imaging ability were characterized by small animal MRI system (Aspect M7). Transendothelial electrical resistance (TEER) was obtained on an epithelial volt-ohmmeter (Millicel-RES, Millipore, USA). The optical absorption in the cellular cytotoxicity experiment was decected by the Microplate Photometer (Multiskan FC, Themo,

USA). Fluorescent images of cells were captured by fluorescence microscope (Ningno yongxin optics co., Ltd. Ningbo, China), and the confocal laser scanning microscopy (Nikon Eclipse Ni-E, Japan). The flow cytometry data was obtained by BD LSRFortessa™ Cell Analyzer.

Synthesis of ultrathin Gd_2O_3 nanodisks. Dissolve 0.75 mmol gadolinium acetate hydrate in a mixed solvent of 1.5 g Oleic acid and 8.13 g Oleylamine under vigorous stirring and vacuum at 120 ℃ for 1h. The resulting solution was heated to 310 ℃ under Ar flow for 4h. After cooling the reaction mixture to room temperature, a 1:4 mixed solution of hexane and acetone was added into the mixture product and the white product was precipitated by centrifugation (12000 rpm, 10 min). The as-prepared Gd_2O_3 nanodisks were dispersible in cyclohexane for subsequent experiments.

Synthesis of G@I-P nanoparticles. 5 mL Gd_2O_3 nanodisks in hexane with the concentration of ~5 mg/mL were added to a dichloromethane solution of $NOBF_4$ (5 mL, 0.01 M) at room temperature. the precipitation of Gd_2O_3 nanodisks can be observed after stirring the mixture. The precipitated Gd_2O_3 nanodisks were subsequently obtained by centrifugation and then redispersed in DMF. Then, Gd_2O_3 nanodisks solution in DMF is slowly added over 5 min into PVP/ H_2N-PEG-NH_2 (55.8 mg/18.6 mg) solution in DMF and was stirred overnight. The surface-modified Gd_2O_3 nanodisks (named G-P) were then purified by precipitation with the addition of toluene, and the precipitated Gd_2O_3 nanodisks were redispersed in 6 mL deionized water for further use.

Then, we mixed $IrCl_3$ solution (25.2 μmol, 6 mL in H_2O) and the Gd_2O_3 nanodisks solution. The mixture solution was dropped into 12 mL of ethanol under magnetic stirring and kept at 25 ° C for overnight. The mixture solution was refluxed at 100 ° C for 6 h and removed the excess solution by rotary evaporation. The obtained products of Gd@Ir-PEG (named G@I-P) was redissolved in H_2O.

Synthesis of G@IT-R nanoparticles. Gd@Ir-PEG modified with RVG29 can be easily obtained by using a heterobifunctional crosslinker. Briefly, 10 mg of MAL-PEG5000-NHS were added into 10 mL of Gd@Ir-PEG (1 mg/mL) in phosphate buffer and kept at 25 °C for 4 h with stirring. Then the resulting solutions were centrifuged and redissolved in phosphate buffer (pH 7.4, 10 mL) containing the RVG29 peptides (1 mg). Afterward, reactions were allowed to continue at the same condition for another 4 h. The final solutions were centrifuged to obtain Gd@Ir-RVG29 (named G@I-R).

For TMB loading, TMB in DMSO (0.05 M, 1 mL) was added dropwise into the above-prepared Gd@Ir-RVG29 in phosphate buffer under ultrasonication for 5 min and stirred under nitrogen atmosphere for 12 h at 4℃. Then, the product was separated from solution by centrifugation and wash with phosphate buffer several times to obtain Gd@Ir/TMB-RVG29 (named G@IT-R). The product was stored at 4 °C for further use.

O_2 Generation of G@IT-R. the oxygen generation of G@IT-R incubated with PBS at different pH containing H_2O_2 (100 μM) was recorded through a portable dissolved oxygen meter.

ABTS Free Radical Scavenging Activity of G@IT-R. The ABTS decolorization assay was utilized to estimate the free radical scavenging capacity of G@IT-R. 2.45 mM $K_2S_2O_8$ mixed with 7 mM ABTS in H_2O for 12 h to produce ABTS radical (•ABTS). Next, the OD (734 nm) of ABTS solution with different concentrations of G@IT-R (AE) and pure •ABTS solution (AA) were recorded. The scavenging efficiency of •ABTS was calculated as follow: [(AA - AE)/AA] * 100.

TMB Oxidation and Photothermal Effect of G@IT-R. G@IT-R (0.5 mg) were respectively dispersed in buffer solution (1 mL) with different pH (4.0, 5.0, 6.5, 7.4, 8.0, and 9.0). After the reaction for 5 min, UV-vis spectra were recorded. For the NIR-II photothermal test, 1 mL G@IT-R solution at 0.5 mg/mL with H_2O_2 (100 μM) was irradiated by 1064 nm laser (1 W/cm^2, 5min) in different pH condition. The photothermal performance curves were monitored every 15 s. And the change of temperature was recorded by the thermal imager.

Cell Culture. GL261 cells, and bEnd.3 cells were plated on culture dishes incubated with DMEM medium supplemented by FBS and 1% antibiotics at 37 °C and 5% CO_2.

***In Vitro* BBB-Crossing Efficiency**：To establish the *in vitro* BBB model, we use the mice brain endothelial bEnd.3 cell. Briefly, we seeded 2 × 10^5 bEnd.3 cells in the transwell upper chamber (0.4 μm pore size) and cultured for about 7 days to reach

TEER higher than 200 Ω cm^2. Then we seeded GL261 cells on the bottom chamber and co-incubation for 24 h. Then we added FITC labeled G@IT-R, G@IT and IT-R into the apical chamber and waiting for 4 h. The GL261 cell's nuclei were stained by DAPI for 10 min, and the Fluorescence intensity of cells in the bottom well was observed by CLSM and quantified by flow cytometry.

Autophagosome evaluation：4×10^6 Pfu of adenovirus expressing Ad-GFP-LC3B were added into the confocal dishes (NEST) containing 10^5 GL261 cells for incubating for 24 h. Then, the culture medium of transfected cells was subsequently replaced with a fresh medium containing the different nanodrugs for further culture. After that, we used Hoechst solution for cell nuclei staining and used CLSM to characterize the changes of the LC3B in GL261 cells treated with different treatments.

***In Vitro* Cell Viability Assessment:** Cells were incubated in 96 well plates with a cell density of 1×10^4 cells/well. After that, cells were applied different treatments and incubated for 24 h. CCK8 assays were used to determine cell viability by analyzing the absorbance at 450 nm. Significance was set as $p < 0.05$ (*), $p < 0.01$ (**) and $p < 0.001$ (***)

Western Blotting: After different treatments, cells washed with PBS were lysed by RIPA Lysis buffer to harvest total proteins. The protein concentration was quantified by using BCA protein assay kits. After adding an equal amount of total protein to each well, the separated proteins were transferred to the PVDF membrane from the SDS-PAGE gradient gel. Then, the membranes were incubated with antibodies

against β-actin (Bioss, Beijing, China) (1:1000), SQSTM1/p62 Rabbit Polyclonal Antibody (Beyotime) (1:1000) or LC3B Rabbit Polyclonal Antibody (Beyotime) (1:1000) at 4 °C overnight, followed by an HRP-conjugated goat anti-mouse immunoglobulin G (IgG) (Bioss, Beijing, China). The protein bands were performed using the enhanced chemiluminescence solution reaction.

Apoptosis Assay: The apoptosis assays were performed to investigate the cell death mechanism with different treatments. Specifically, GL261 cells treated with respective treatments were washed with PBS several times and then treated with annexin V-FITC/propidium iodide (PI) apoptosis detection kit. Finally, the cells were detected by the flow cytometer.

Animals: All of the animal experiments were conducted according to the rules of the Institutional Animal Care and Use Committee of Tsinghua University (IACUC, 20200330005). Female C57BL/6J mice (6-8 weeks) were used to establish the intracranial GBM mouse model. Specifically, 1.5×10^5 GL261 cells in 5μL PBS were injected into one mouse brain (0.6 mm anterior to the bregma, 1.8 mm lateral; depth of 2 mm).

***In Vivo* Tumor Imaging and Biodistribution.** The mice bearing orthotopic glioma were i.v. injected with G@IT-R in PBS (10 mg Gd/kg). Then mice were imaged by a small animal MRI system at different preset time points of post-injection. Mice were sacrificed after 12, 24, and 48 h post-injection, respectively. The major organs

together with tumor tissue were harvested, weighed, and stored at −80 °C before ICP-AES analysis of Gd element for the biodistribution evaluation of G@IT-R.

***In Vivo* Therapeutic Effect**: To evaluate the PTT therapeutic effect of G@IT-R, the mice randomized into six groups were tail vein injected with 100 μL of different nanodrugs (dose: 3 mg Gd/kg or 1 mg Ir/kg). Laser irradiation was carried out at 12 h p.i. Based on the results of temperature decay with tissue depth *in vitro*, 1064 nm laser parameters of 1 W/cm^2 for 10 min were adopted.

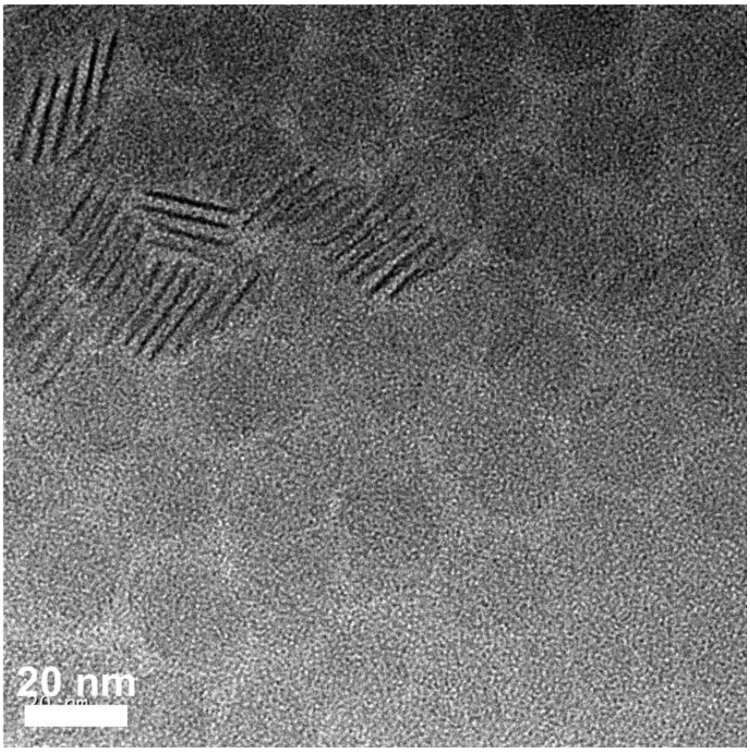

Figure S1. TEM image of as-synthesized Gd_2O_3 nanodisks.

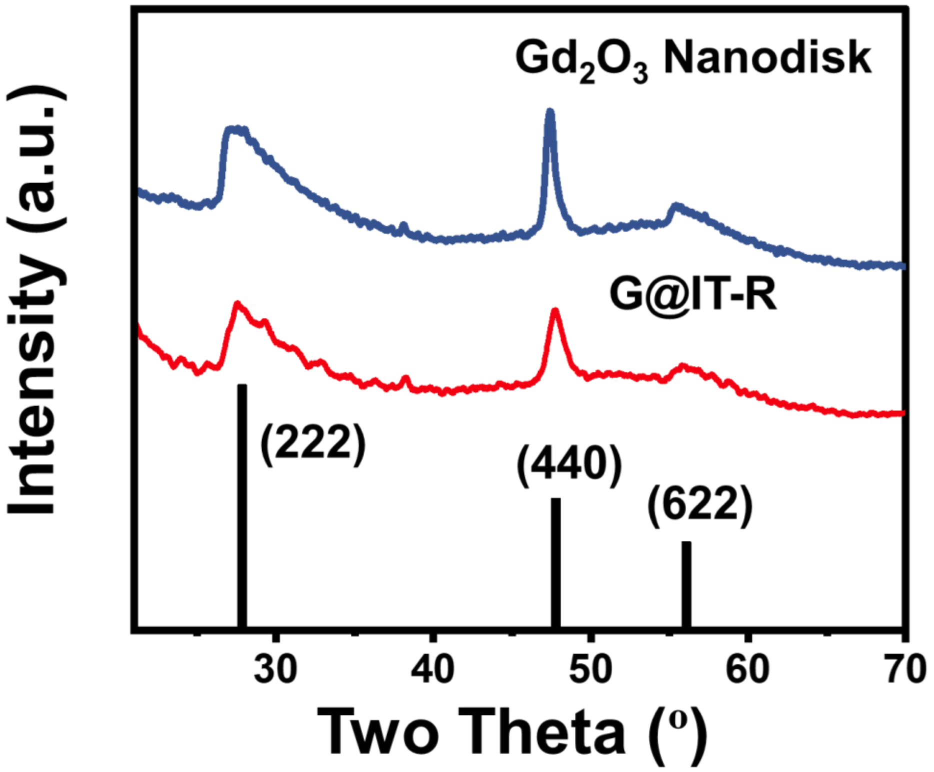

Figure S2. XRD patterns of Gd_2O_3 nanodisks and G@IT-R nanomachines.

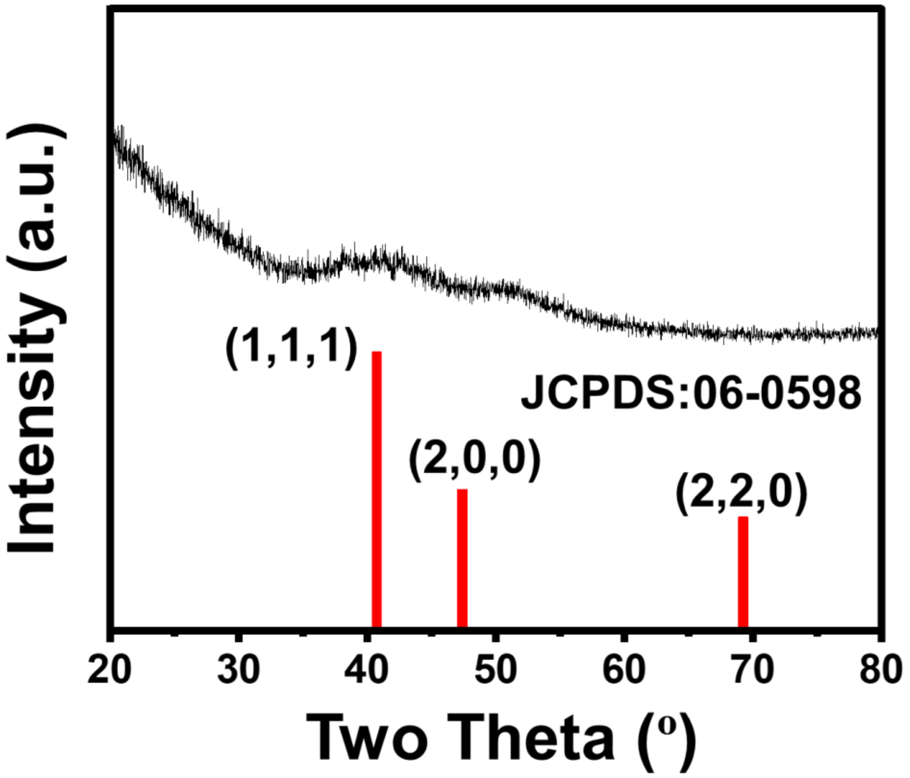

Figure S3. XRD pattern of Ir nanodots.

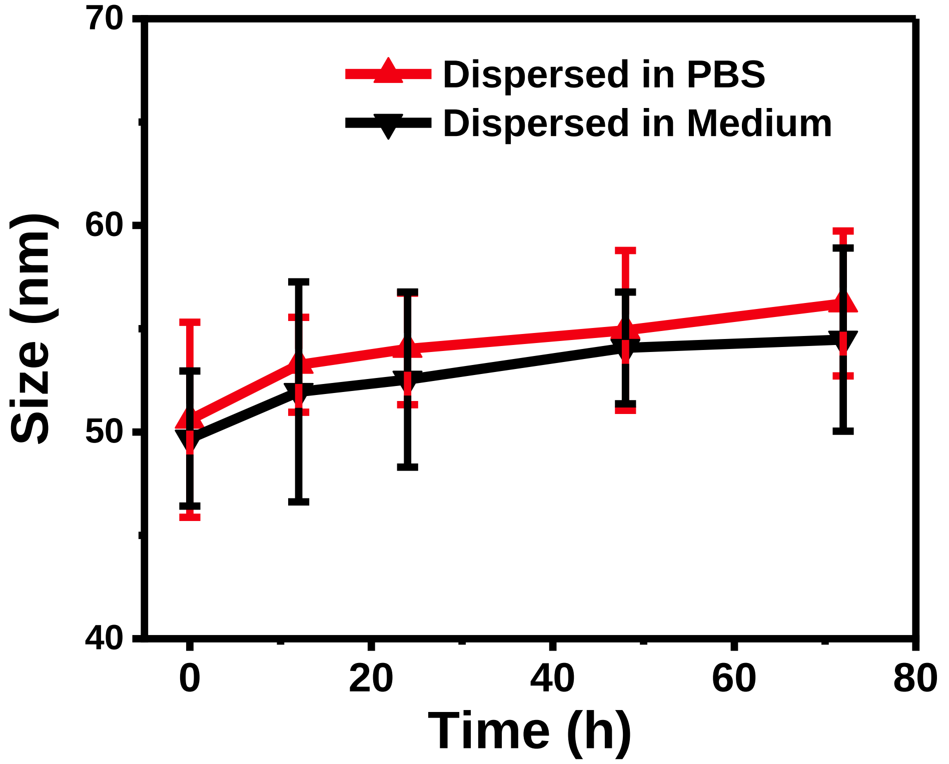

Figure S4. Changes in hydrodynamic size of G@IT-R suspended in PBS buffer and 10% fetal bovine serum (FBS) complete medium, respectively.

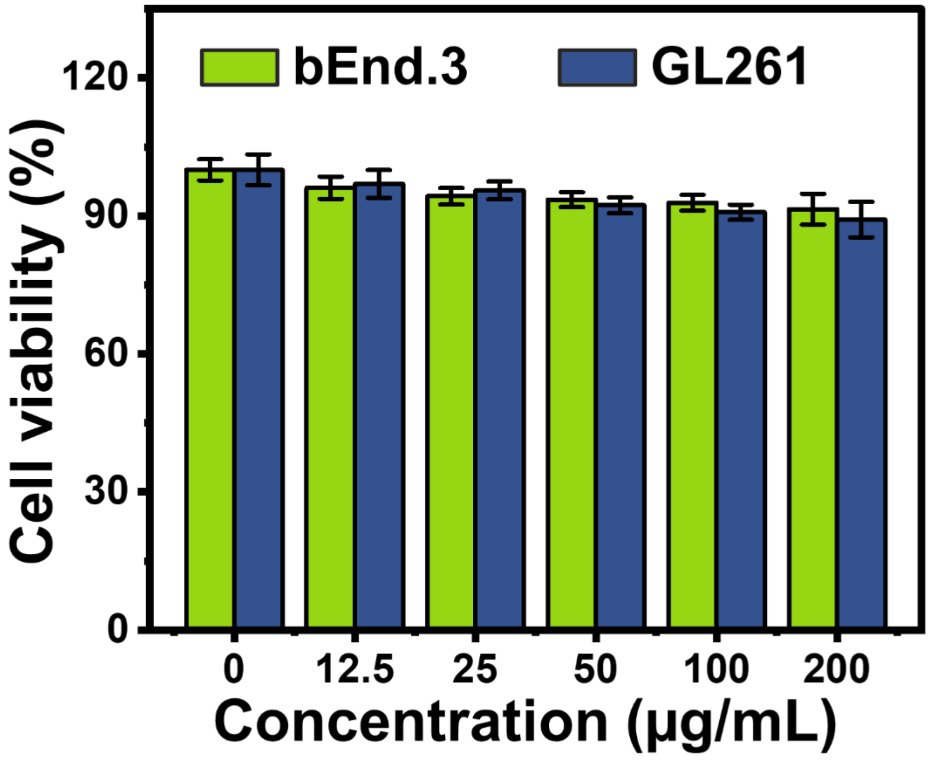

Figure S5. Cell viabilities of bEnd.3 and GL261 incubated with G@IT-R at different concentrations. Data are expressed as mean ± SD (n= 6).

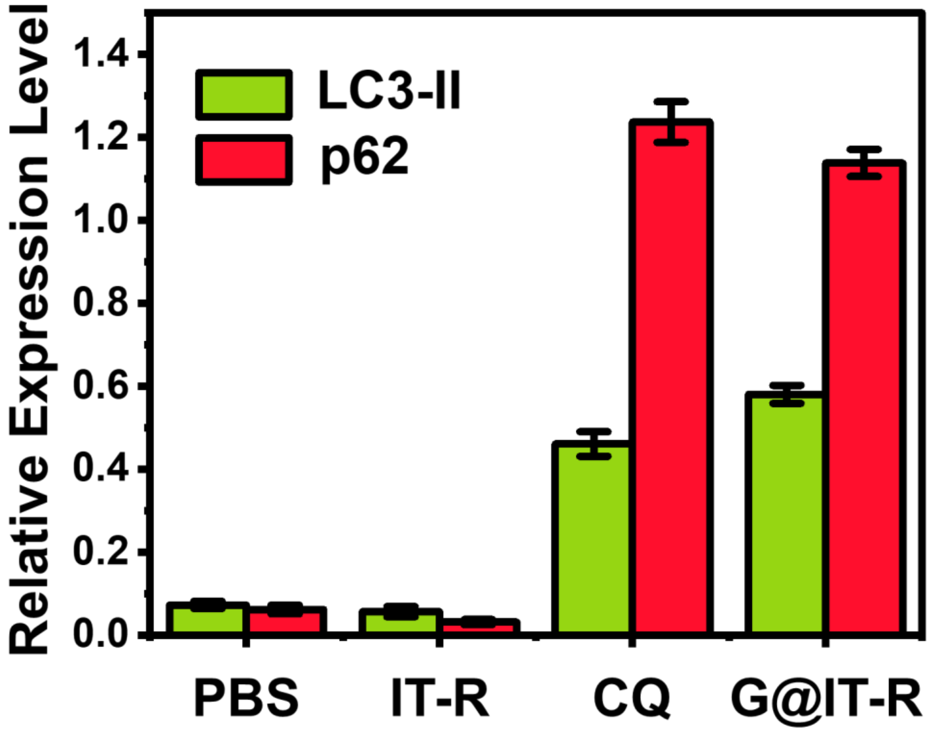

Figure S6. Quantification of protein expression levels of LC3-II and p62 using Western blot analysis.

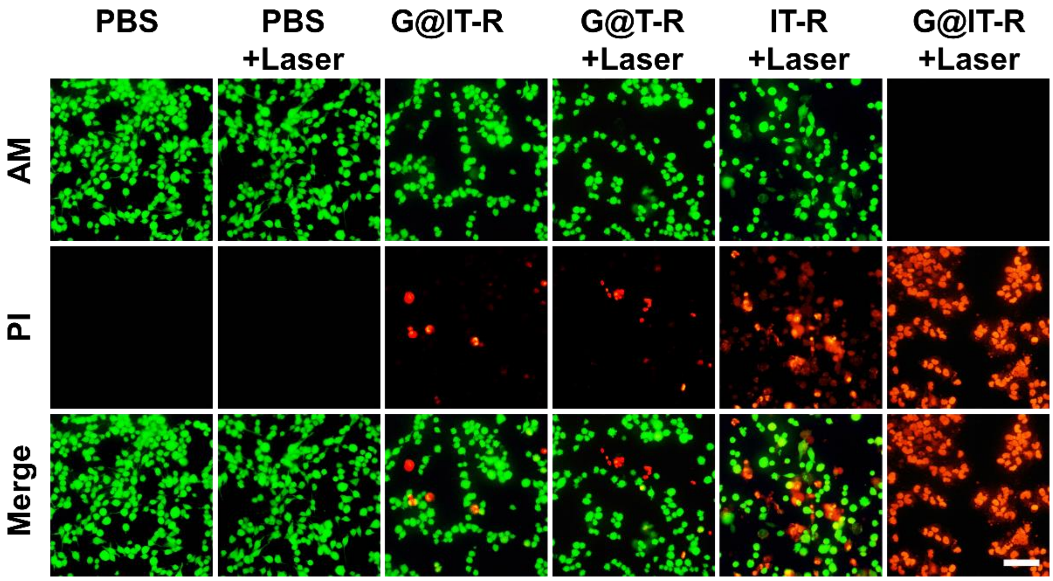

Figure S7. FL images of GL261 cells after different treatments. (Viable cells were stained green with Calcein-AM, and dead/later apoptosis cells were stained red with PI. Scale bar: 100 μm.)

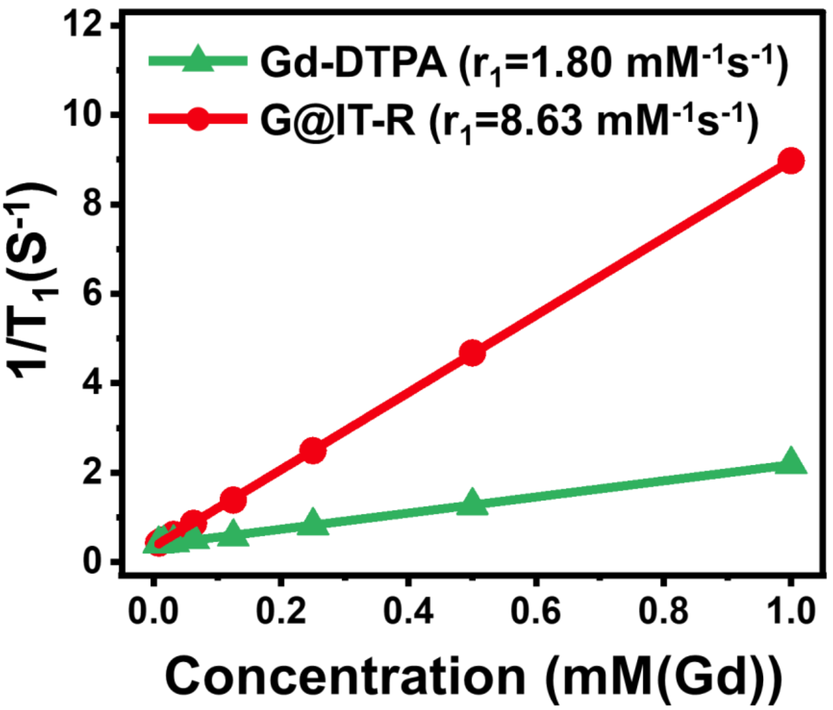

Figure S8. The r_1 relaxivities of Gd-DTPA and G@IT-R.

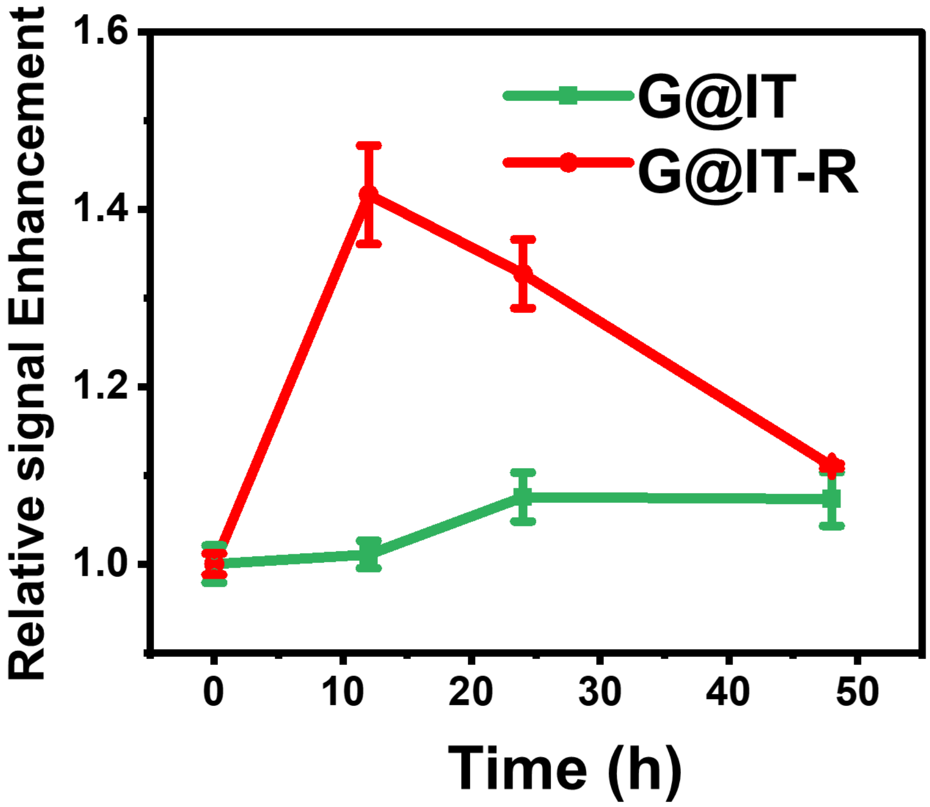

Figure S9. Graphs show the measured relative signal enhancement after injection of G@IT or G@IT-R NPs at different time points.

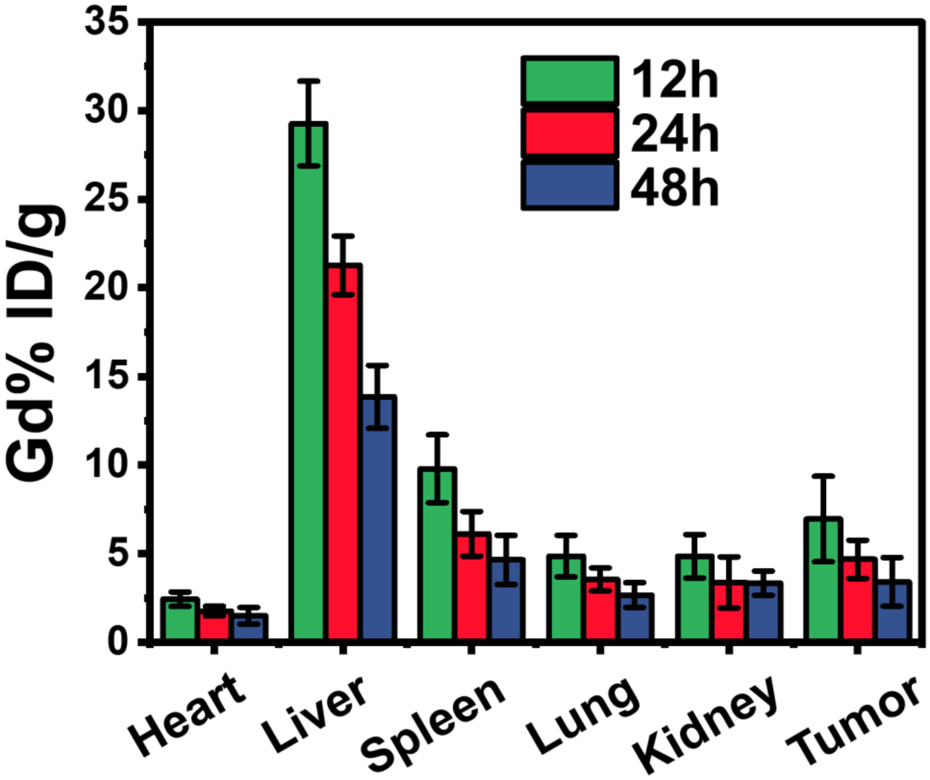

Figure S10. The body distribution of Gd content after the intravenous injection of G@IT nanomachines at different time points.

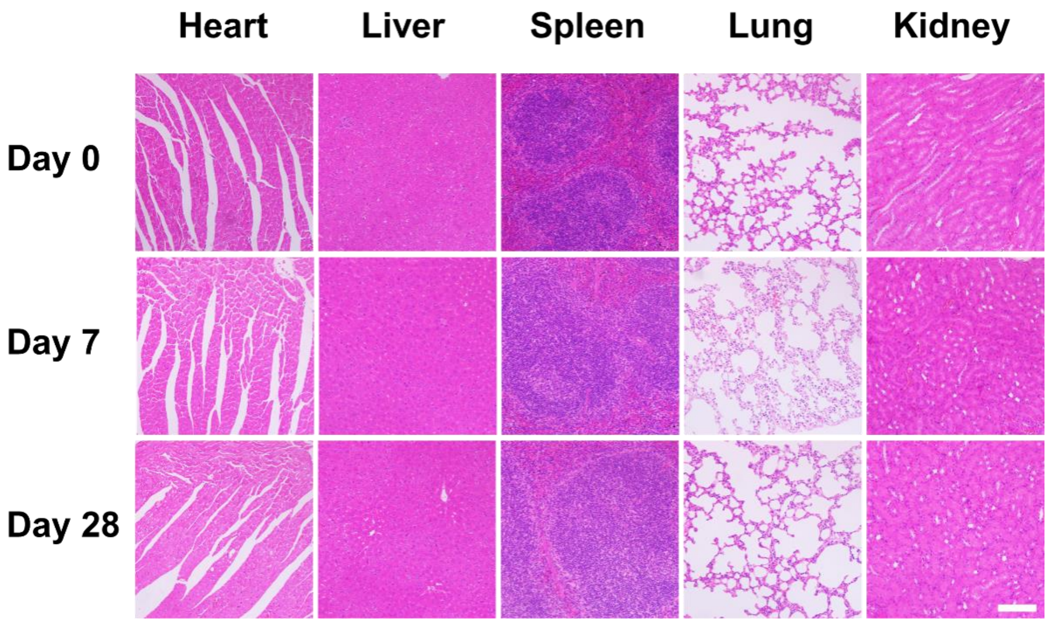

Figure S11. H&E-stained slice images of main organs at different time points from mice treated with G@IT-R. Scale bar:100 μm.

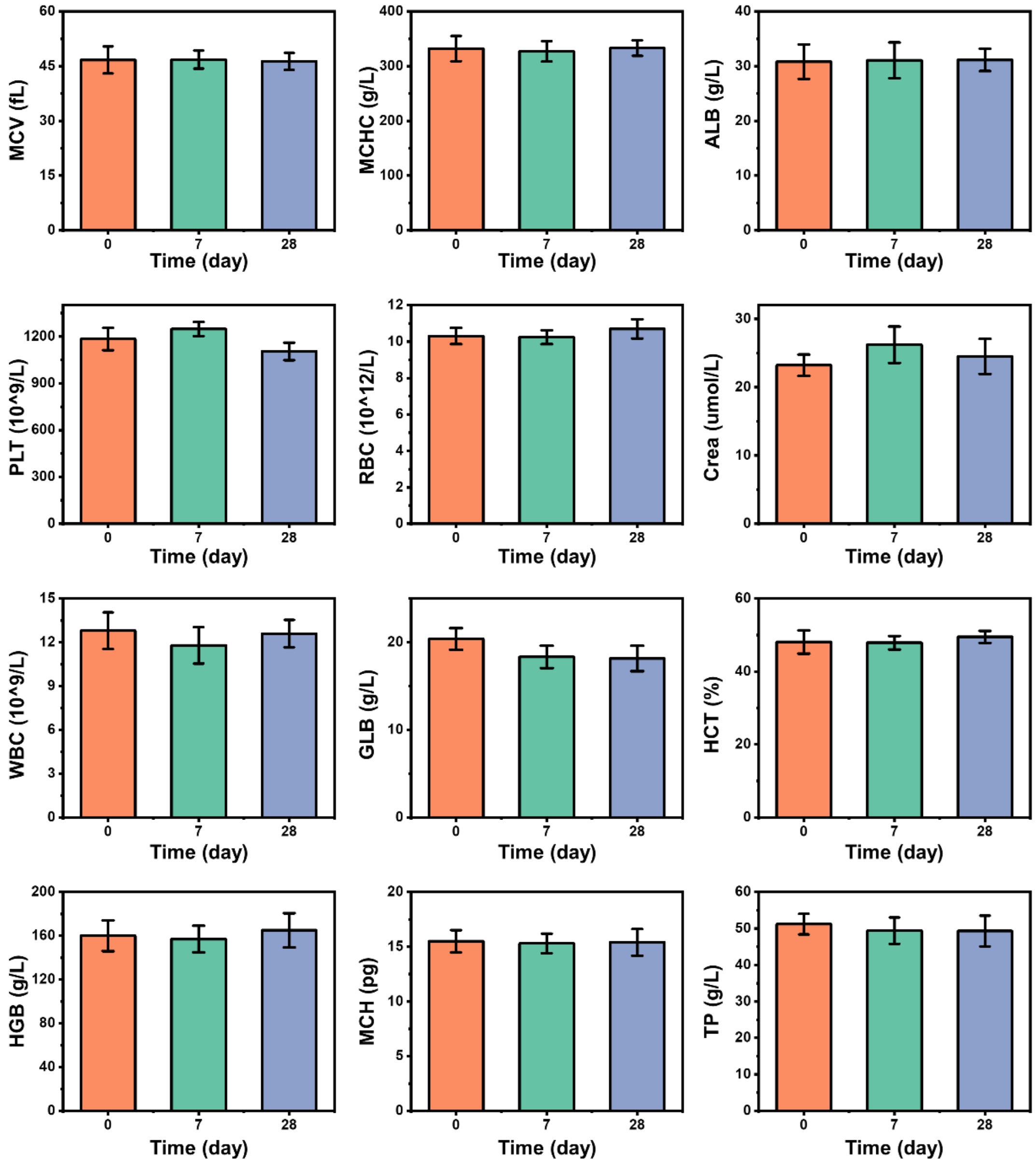

Figure S12. Blood biochemistry at different time points of healthy female C57BL/6J mice treated with G@IT-R. Erythrocyte mean corpuscular hemoglobin concentration (MCHC), Hematocrit (HCT), Red blood cells (RBC), Erythrocyte mean corpuscular volume (MCV), Platelet (PLT), Erythrocyte mean corpuscular hemoglobin (MCH), White blood cells (WBC), Haemoglobin (HGB), Globulin (GLB), Creatinine (Crea), Albumin (ALB) and Total Protein (TP). Data are represented as mean ± SD (n = 3).
